# Investigating the Processing Potential of Ethiopian Agricultural Residue Enset/*Ensete ventricosum* for Biobutanol Production

**DOI:** 10.3390/bioengineering9040133

**Published:** 2022-03-24

**Authors:** Nebyat Seid, Pia Griesheimer, Anke Neumann

**Affiliations:** 1 Technical Biology, Institute of Process Engineering in Life Science 2, Karlsruhe Institute of Technology, 76131 Karlsruhe, Germany; nebyat.seid@kit.edu; 2 Institute of Catalysis Research and Technology, Karlsruhe Institute of Technology, 76344 Eggenstein-Leopoldshafen, Germany; pia.griesheimer@kit.edu

**Keywords:** enset biomass, biobutanol, *C. saccharoperbutylacetonicum DSM 14923*, ABE fermentation

## Abstract

The Enset plant is a potential food source for about 20 million Ethiopians. A massive amount of residual byproduct is discarded from traditional Ethiopian Enset food processing. This study shows a compositional analysis of Enset biomass and its use for biobutanol production. The Enset biomass was pretreated with 2% (*w*/*v*) NaOH or 2% (*v*/*v*) H_2_SO_4_ and subjected to enzymatic hydrolysis. The enzymatic hydrolysates were then fermented anaerobically by *C. saccharoperbutylacetonicum DSM 14923*. The majority of Enset biomass waste samples contained 36–67% cellulose, 16–20% hemicelluloses, and less than 6.8% lignin. In all alkali-pretreated Enset biomass samples, the enzyme converted 80–90% of the biomass to glucose within 24 h, while it took 60 h to convert 48–80% of the acid-pretreated Enset biomass. In addition, the alkali pretreatment method released more glucose than the acid pretreatment in all Enset biomass samples. After 72 h of ABE fermentation, 2.8 g/L acetone, 9.9 g/L butanol, and 1.6 g/L ethanol were produced from mixed Enset waste hydrolysate pretreated with alkali, achieving an ABE yield of 0.32 g/g and productivity of 0.2 g × L^−1^ × h^−1^, showing the first value of butanol produced from Enset biomass in the literature.

## 1. Introduction

Currently, most industrial and transport sectors rely on petroleum fuels as their main source of energy [1]. However, researchers predict that the global supply of petroleum fuels will be depleted by 2070–2080. In addition, the consumption of petroleum products contributes to global warming and environmental pollution [2]. In developing countries like Ethiopia, the scarcity of energy increases poverty and unemployment [3], and over 80% of energy needs are met by hydropower and biomass production [4]. Moreover, 75% of foreign earnings are spent on importing petroleum fuels [5]. In this context, the rising demand for energy on the planet and our pressing environmental problems can be addressed by better utilizing biofuels [6].

Biobutanol (C_4_H_9_OH) holds great promise as a biofuel for the next generation. Compared to bioethanol, it produces higher energy per gallon, with a greater heat of combustion and higher-octane numbers. Above all, it mixes better with gasoline without adapting the gasoline engine and is safe to use due to its lower vapor pressure [7,8,9]. Biobutanol can be produced from sugar, starch, or certain food crops [10], but the production process creates food and energy competition problems primarily due to sudden climate changes, including dry seasons and flooding [11]. Researchers have suggested that lignocellulosic biomass could represent a promising raw material for biobutanol production because of its abundance, high availability, renewability, and versatility [12]. Biobutanol has been produced from lignocellulosic biomass such as barley straw [13], corn stover [14], wheat straw [15], rice hull [16], and sugar cane bagasse [17]. However, the main obstacle to using lignocellulosic biomass as a raw material for biobutanol production is the higher proportion of lignin, which firmly binds cellulose and hemicelluloses, and cannot be used by microorganisms [18]. For the majority of agricultural biomass, the lignin mass fraction is around 10–25% [19], though the lignin mass fraction for Enset biomass is estimated to be lower. Berhanu et al. [20] estimated that the low lignin contents of the Enset fiber and the inflorescence stalk main are 10.53% and 5.72%, respectively. Further going in its favor, the Enset plant is well adapted to growing in many soil conditions, has favorable growth measurements (generally 4–8 m high, sometimes reaching up to 11 m), and high drought resistance [21,22]. The Enset plant could thus represent a promising source of lignocellulosic biomass for biobutanol production.

The Enset plant (*Ensete ventricosum* [Welw.] Cheesman) (false banana) is a herbaceous monocarpic plant in the Musaceae family [23]. It is similar to the banana plant in that it has an underground corm, a bundle of sheaths, and large leaves, but the seedy fruit from the Enset plant is not edible like a banana [24]. The Enset plant is composed of multiple components; the ratio of each component varies with the varieties of the plant. Nurfeta et al. [25] estimated the ratios of its components to be in the range of 6–16% lamina, 4–21% midribs, 46–60% pseudostem, and 10–30% corm. The Enset plant is a potential food source for about 20 million Ethiopians [26]. In addition, in Uganda, Enset plant parts are used for therapeutic purposes and local beer brewing [27]. A recent study predicted that the crop can be grown more and provide food for an additional 87.2 to 111.5 million Ethiopians, with the potential to expand its farming into sub-Saharan Africa [28]. Kocho, bulla, and amicho are foods made from Enset plants by scraping and fermenting leaf sheaths and corm [29]. In traditional Ethiopian Enset food processing, a massive amount of residual byproduct is discarded; only the Enset fibers from this waste are used, to make bags, ropes, mats, and sieves [20]. In a recent study, Erebo [30] attempted to assess the processing potential of Enset wastes for ethanol production, and showed that ethanol can be produced. The main steps to produce biobutanol from lignocellulosic biomass are pretreatment, enzyme hydrolysis, and ABE fermentation [31]. To date, no comprehensive study has been carried out to evaluate the production process of biobutanol from Enset plant biomass. This study aimed to analyze the composition of the Enset biomass and examine the processing potential for biobutanol production.

## 2. Materials and Methods

### 2.1. Raw Materials and Sample Preparation

**Figure 1:**
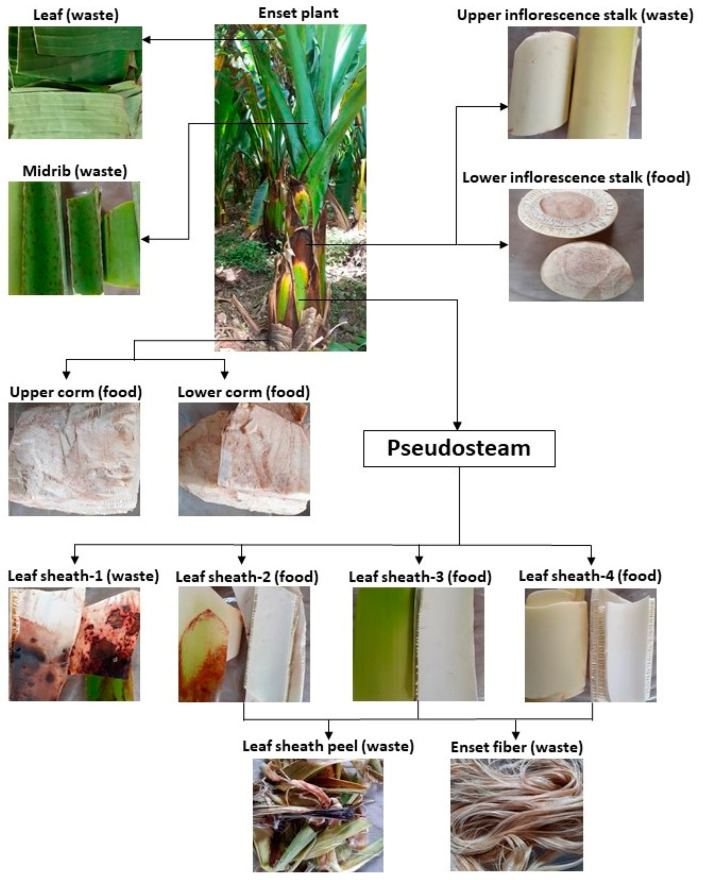
Morphology of Enset biomass based on traditional Enset food processing. (This photo was taken by one of the authors at a private Enset plantation in Wolkite, Ethiopia).

The samples of Enset biomass were collected from a privately owned Enset plantation in Wolkite, Ethiopia. The Enset plant was selected at random and separated into different parts based on traditional Enset food processing: leaf sheath layers (leaf sheath-1 (LS1), leaf sheath-2 (LS2), leaf sheath-3 (LS3), and leaf sheath-4 (LS4)), upper inflorescence stalk (UIS) and lower inflorescence stalk (LIS), upper corm (UC) and lower corm (LC), leaf sheath peel (LSP), Enset fiber (EF), midrib (M), and leaf (L) (Figure 1). Samples were manually chopped into smaller 3–6 cm pieces using a stainless-steel knife. The materials were dried separately in the sun for 4–5 days, pulverized with the knife mill, and sieved with various sieve sizes. The dry powder material was stored in a plastic bag at room temperature. The sample powder was subjected to composition analysis and pretreatment. The chemicals used in this study were purchased from Sigma-Aldrich Chemie GmbH (Hamburg, Germany) or Carl Roth GmbH + Co. KG (Karlsruhe, Germany).

### 2.2. Compositional Analysis

The compositions of the Enset biomass parts were analyzed. The acid detergent fiber (ADF), acid detergent lignin (ADL), and neutral detergent fiber (NDF) of the sample were determined by Gesellschaft für Analysentechnik HLS using the ANKOM technology method (ANKOM^A2000^ fiber analyzer) [32,33,34] at the University of Hohenheim, Stuttgart, Germany. Based on the van Soest method [35], the cellulose content of the sample was determined by subtracting ADL from ADF; the difference between NDF and ADF gives the hemicellulose content, and the lignin content was acid detergent lignin (ADL) [36]. The elemental analysis which is carbon, hydrogen and nitrogen content, moisture content, ash content, and calorific value of the Enset biomass samples were determined at the Karlsruhe Institute of Technology (KIT), Institut für Katalyseforschung und-technologie (IKFT) according to the ISO 16948: 2015, ISO18134, ISO 18122, and ISO 181251 protocols, respectively. Conversion of the results to a dry reference condition was carried out with the respective analytical moisture contents of the samples. Thus, the parameters of the ash content, calorific value, carbon, and nitrogen were corrected upward. An exception was hydrogen, for which a downward correction was made since the H component from the water (moisture) must first be subtracted from the measured value of the total hydrogen. Waste samples with a high cellulose content were selected for analysis of the monomeric sugar and degradation products of the biomass; analyses were performed according to the National Renewable Energy Laboratory (NREL/TP-510-42623) standard using the two-step hydrolysis method [37]. The monomeric sugar results were corrected with the respective sugar recovery standards.

### 2.3. Pretreatment and Enzymatic Hydrolysis

The following samples were selected for pretreatment and enzymatic hydrolysis experiments: leaf sheath peel, Enset fiber, midrib, and mixed Enset waste, which was a mixture of leaf sheath, upper inflorescence stalk, leaf sheath peel, Enset fiber, midrib, and leaf. Then, 20 g dried and milled (1 mm particle size) samples were placed in a 500 mL Erlenmeyer flask, mixed with 200 mL 2% (*w*/*v*) NaOH or 2% (*v*/*v*) H_2_SO_4_. The mixture was autoclaved at 121 °C for 20 min. At the end of the autoclave cycle, the samples were cooled, centrifuged at 4700× *g* for 30 min, and filtered. The filtrate was analyzed for sugars and degradation products. The residue was washed repeatedly with 2 L deionized water, and the pH was adjusted to 5 before it was filtered again using a muslin cloth [38]. According to the NREL (NREL/TP-510-42621) standard, residue samples were dried at 105 °C for 24 h using a convection oven to determine their moisture content [39], and subjected to enzymatic hydrolysis.

The enzymatic hydrolysis experiment was performed in a 500 mL Erlenmeyer flask, where 5 g (dry weight) alkali- or acid-pretreated biomass was mixed with 100 mL liquid containing 28 FPU/g cellulase enzyme (Cellic CTec2) (Sigma-Aldrich Chemie GmbH, Hamburg, Germany) and 9.6 g/L citrate buffer (pH 5.0) to keep the pH at 5. The experiment was conducted at 50 °C and 130 rpm (Infors Thermotron, Infors AG, Bottmingen, Switzerland) for 72 h [40]. A 1.5 mL aliquot was withdrawn every 12 h, chilled on ice, centrifuged at 10,000 rpm for 10 s, and the glucose concentration was measured. For the control experiment, a wheat straw sample and blank (enzyme without substrate) were used. All experiments were performed in triplicate. The glucose concentration was corrected by subtracting the respective blank controls.

### 2.4. Microorganism and Culture Maintenance

*Clostridium saccharoperbutylacetonicum DSM 14923* was acquired from the German Collection of Microorganisms and Cell Cultures (DSMZ, Braunschweig, Germany). The preculture medium was tryptone-glucose-yeast extract (TGY), which contained 30 g/L tryptone, 20 g/L glucose, 10 g/L yeast extract, and 1 g/L cysteine-HCl·H_2_O. The strain was regularly maintained in 50% glycerol stocks at −80 °C [41]. The glycerol stocks were prepared according to the method described by Infantes et al. [42], whereby 5 mL culture grown for 13–14 h at 30 °C was placed in a Hungate-type (sterilized and anaerobized) tube, and centrifuged at 3000× *g* and 4 °C for 5 min. The supernatant was removed from the tube, and 1 mL equal volumes of culture medium and 50% (*v*/*v*) glycerol solution were added to the pellet and frozen at −80 °C. For cultivation, 1 mL glycerol stocks were anaerobically revived in a 118 mL serum bottle (Glasgerätebau Ochs, Bovenden, Germany) with 50 mL TGY medium, until the optical density (OD) at 600 nm reached 1.0–2.0.

### 2.5. ABE Fermentation

**Figure 2:**
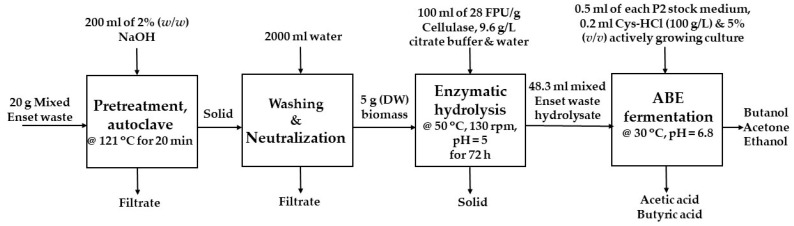
Biobutanol production process from mixed Enset waste.

ABE fermentation was performed with mixed Enset waste hydrolysate as a carbon substrate, prepared by enzymatic hydrolysis after alkali pretreatment, and supplemented with 1% (*v*/*v*) P2 stock medium. The latter contained buffer stock solution (50 g/L KH_2_PO_4_, 50 g/L K_2_HPO_4_, and 220 g/L CH_3_COONH_4_), mineral stock solution (20 g/L MgSO_4_·7H_2_O, 1 g/L MnSO_4_·H_2_O, 1 g/L FeSO_4_·7H_2_O, and 1 g/L NaCl), and vitamin stock solution (0.1 g/L para-aminobenzoic acid, 0.1 g/L thiamin, and 0.001 g/L biotin) [43]. Next, 48.3 mL hydrolysate was mixed with 1 g/L yeast extract and 1 g/L resazurin, and the pH was adjusted to 6.8 with NaOH/H_3_PO_4_ [44]. The medium was then poured into 250 mL serum bottles, which were sealed with a rubber stopper and an aluminum cap, and anaerobized. The anaerobization process was carried out by flashing the bottles with a mixture of 20% CO_2_ and 80% N_2_ gas using needles connected to the gas lines. After the anaerobization process, 0.2 mL Cys-HCl (100 g/L) was added to the bottles using syringes and needles, and then autoclaved. After autoclaving, 0.5 mL each of sterile-filtered and anaerobic P2 stock solutions was added. The bottles were inoculated with 5% (*v*/*v*) actively growing culture and incubated for 72 h at 30 °C (Infors Thermotron, Infors AG, Bottmingen, Switzerland) [44]. During the fermentation, 1 mL of a sample was taken for analysis within 8 h, and the pH value of the sample was measured without controlling it. For the control experiment, 40 g/L glucose solution was used as a substrate. All experiments were carried out in triplicate. The overall biobutanol production process from mixed Enset waste is illustrated in Figure 2.

### 2.6. Analytical Methods

Cell growth was measured by taking absorbance measurements (OD_600_) using an Ultrospec 1100 pro spectrophotometer (Amersham Biosciences, Uppsala, Sweden), and Profilab pH 597 (Xylem Analytics, Weilheim, Germany) was used for pH measurement. The monomeric sugars, degradation products, and fermentation metabolites in the samples were analyzed by high-performance liquid chromatography (HPLC) in an 1100 Series System (Agilent Technologies, Waldbronn, Germany), with the column model a Rezex ROA-Organic Acid H^+^ (8%) and with 5 mM sulfuric acid eluent, as described by Stabel et al. [45]. The method was modified with a column temperature of 55 °C and an eluent flow rate of 0.6 mL/min to detect furfural and hydroxymethylfurfural (HMF) [46]. Butyric acid was analyzed separately using the reversed-phase column Synergi™ 4 μm Fusion-RP 80 Å (150 mm × 4.6 mm) (art. No. 00F-4424-E0, Phenomenex Inc., Aschaffenburg, Germany) at a 30 °C column temperature, with eluent compound 20 mM KH_2_PO_4_, at pH 2.5, and a flow rate of 1 mL/min [47]. The acetone pick in HPLC was overlapped with the butyric acid pick; hence, acetone was analyzed with headspace-gas chromatography (GC) using a 6890 N Network GC-System (Agilent Technologies Deutschland GmbH, Waldbronn, Germany) equipped with a flame ionization detector (FID). The chromatographic column was an Agilent FFAP, with capillary as the stationary phase (30.0 m × 320 μm × 0.50 μm nominal). The carrier gas was helium at 1 bar and 3.2 mL/min. The acetone pick was separated by a temperature gradient that was initially held at 40 °C for 2 min, raised at 20 °C/min to 180 °C, and held for 3 min. The headspace GC sample was prepared by adding 100 μL sample into 10 mL serum bottles sealed with butyl septa and screw caps; these contained 0.5 g NaCl, 100 μL 20% (*v*/*v*) H_3_PO_4_, and 100 μL 1-propanol as the internal standard. The bottles were heated to 95 °C for 1 h. A gas-tight syringe was used to withdraw 0.5 mL samples from the gas phase and place these into the GC [48].

## 3. Results

### 3.1. Compositional Analysis

The ash content, moisture content, elemental analysis, and calorific value of the Enset biomass parts are shown in Table 1. The carbon content of the Enset biomass ranged from 37.1 to 42.8%. The majority of the Enset biomass parts had a lower carbon content than the Enset fiber and the leaf, i.e., 41.2% and 42.8%, respectively. The Enset fiber had a relatively similar carbon content to barley straw, at 40.69% [49], but this was lower than that of wheat straw, at 45.58% [50]. The hydrogen content ranged from 5.2 to 6.2%, similar to what is found in most lignocellulose biomasses [49]. The nitrogen was relatively low (<2.5%) in most parts of the Enset biomass except for the leaf (3.3%), though this leaf content would have little impact on the environment during the thermochemical process [51]. Then, the calorific value of most of the Enset biomass parts varied from 14.3 MJ/kg to 17.4 MJ/kg, which was lower than that of banana leaves, at 19.8 MJ/kg [52]. Studies have shown that the biomass used in thermochemical processes must have a calorific value between 17.0 and 22.0 MJ/kg [53]. As it falls below this range, Enset biomass could not be recommended for thermochemical processes. We also noted that the ash content of the Enset biomass parts varied from 4.7% to 19.1%, which was relatively high compared to other lignocellulosic biomasses [54].

Table 2 lists the lignocellulosic composition of Enset biomass parts. The results show that the Enset fiber had a high cellulose content of 67.1%, followed by the midrib and leaf sheath peels, at 40% and 34.1%, respectively. In contrast, the upper and lower corm had lower cellulose contents of 2.2% and 3.8%, respectively. The cellulose content of Enset fiber was higher than those of *Pandanus amaryllifolius* fiber (48.8%) [56], wheat straw (34.6%) [51], barley straw (33.25%) [57], corn stover (31.32%) [58], and sugarcane bagasse (54.87%) [59], but quite similar to banana fibers (60–65%) [60] and pineapple leaf fiber (62.5%) [61]. The majority of the Enset biomasses had less than 20.4% hemicellulose, except for the leaf, which contained 27%. Furthermore, the Enset biomass parts contained less than 6.8% lignin, which was significantly less than that of most lignocellulosic biomasses, which typically have a lignin content of 14–25% [62]. Our findings lead us to propose that Enset biomass can be used to produce biofuels, especially from its fibrous parts, which are an excellent source of fermentable sugars due to their high cellulose content, i.e., the main source of glucose. In addition, the low lignin content of the Enset biomass makes it easier for the pretreatment process to release more fermentable sugars [63].

Complete hydrolysis of the cellulose and hemicellulose contents of the biomass is necessary to determine the amount of monomeric sugar in the biomass. Monomeric is key as oligomeric sugars may further break down into other compounds on hydrolysis with concentrated sulfuric acid [37]. Table 3 shows the composition of sugars and degradation products we found in Enset biomass parts hydrolysate. The cellobiose content of all samples was low 0.8–2.5% (*w*/*w*), indicating that the oligomeric sugars were completely converted to monomeric forms [64]. High percentages of glucose were found in the Enset fiber (65.4% *w*/*w*) and leaf sheath peel (56.4% *w*/*w*) compared to the Midrib (39.1% *w*/*w*) and mixed Enset waste (45.0% *w*/*w*). However, the amount of arabinose in the Enset fiber was significantly lower, at 0.93% (*w*/*w*), than other samples in the range of 2.35–3.28% (*w*/*w*). Other sugars, including xylose, mannose, and galactose, all had similar amounts of between 10.5% and 12.7% (*w*/*w*), which were found across all samples. The acetic acid content of Enset biomass samples ranged from 5.0 to 9.0% (*w*/*w*), while the formic acid was less than 2.24% (*w*/*w*). All samples contained low levels of furfural, between 0.43 and 1.03% (*w*/*w*), and HMF was not detected. In this study, further degradation of the hemicellulose to organic acids and a small amount of furfural was observed. The formation of high levels of acetic acid could be due to xylose degradation [65].

### 3.2. Effect of Dilute Alkali and Acid Pretreatment Method on Enzymatic Hydrolysis

**Figure 3:**
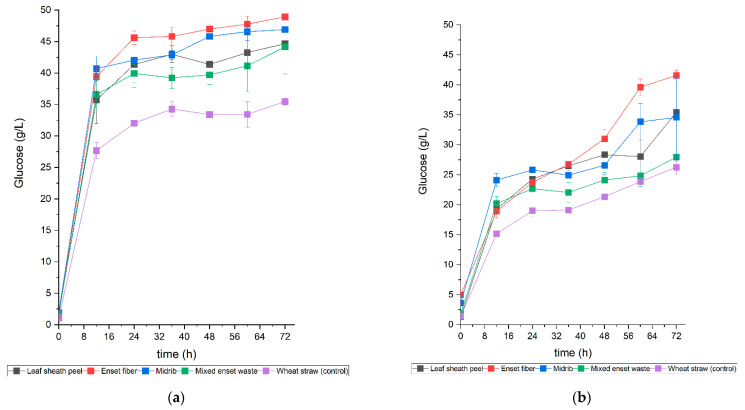
Glucose produced from enzymatic hydrolysis of Enset biomass samples with 5% (dry weight) solid loading, pretreated using different pretreatment methods: (**a**) alkali pretreatment method; (**b**) acid pretreatment method.

In this study, an enzymatic hydrolysis experiment was performed to evaluate the effect of the dilute alkali or acid pretreatment method on the glucose release from each Enset biomass part. Figure 3 shows the glucose concentration results produced from alkali- or acid-pretreated biomass after enzyme hydrolysis with 5% (dry weight) solid loading. In samples pretreated with alkali (Figure 3a), after 36 h, the Enset fiber contained 45.8 g/L glucose, while in midrib, the same amount of glucose was found after 48 h. A similar amount of glucose (44 g/L) was found in leaf sheath peel and mixed Enset waste after 72 h. However, in samples pretreated with acid (Figure 3b), after 72 h, the Enset fiber glucose level was reduced to 41.5 g/L and the converted glucose from midrib and leaf sheath peel was slightly higher than that of the mixed Enset waste, which was showing as 35 g/L. Overall, 24 h was sufficient to convert 80 to 90% of all alkali-pretreated Enset biomass samples to glucose, while it took 60 h to convert 48 to 80% of the acid-pretreated Enset biomass. One possible explanation could be the influence of the pretreatment process on the structural properties of the Enset biomass. One study showed that despite the enzyme mechanism, various factors influence the enzymatic hydrolysis of lignocellulosic biomass, such as the physical, chemical, and morphological properties of the materials [66]. Zhang et al. [67] investigated the effect of structural features of biomass on enzymatic hydrolysis and found that, in addition to the lignin content, the crystallinity of the biomass was an important factor in reducing the enzyme hydrolysis rate. According to this study, for samples with a low lignin biomass, those with high biomass crystallinity took longer to complete enzymatic hydrolysis than samples with low biomass crystallinity [67]. Even though the lignin content of Enset biomass was low, acid-pretreated samples did not necessarily lose their crystallinity. It is important to conduct several tests on the structural properties of Enset biomass before and after pretreatment to better understand this material. In our research, with both methods, the glucose concentration in all Enset biomass samples was higher than in wheat straw samples (control), except for the acid-pretreated mixed Enset waste hydrolysate, which had a similar concentration to the acid-pretreated wheat straw hydrolysate.

**Figure 4:**
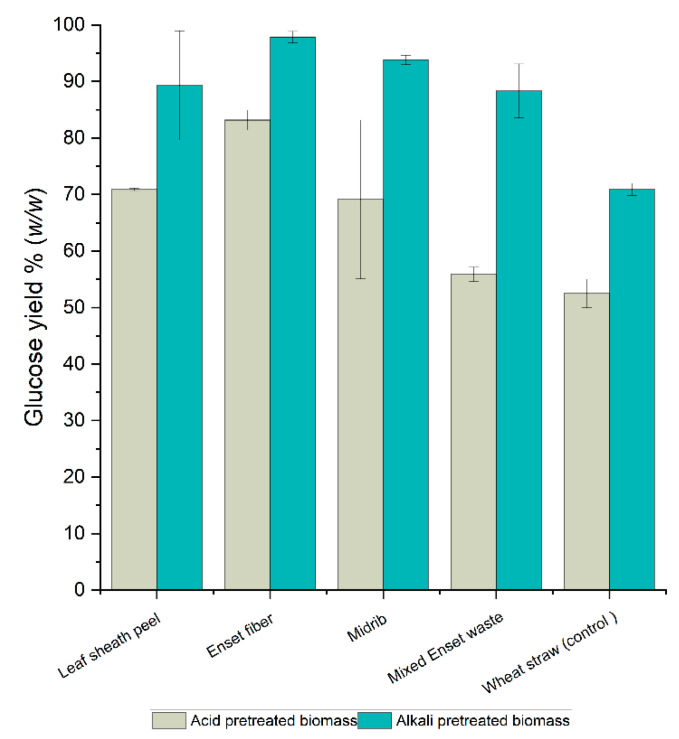
Comparison of the glucose yield after 72 h of enzymatic hydrolysis for acid- and alkali-pretreated Enset biomass samples.

A comparison of the glucose yield for Enset biomass samples pretreated with dilute acid and alkali after 72 h of enzymatic hydrolysis is shown in Figure 4. The percentage of glucose yield was calculated from each pretreated Enset biomass sample. The difference between the acid- and alkali-pretreated Enset fiber and leaf sheath peel yields was less than 20% (*w*/*w*). However, in the mixed Enset waste and midrib samples, the alkali-pretreated samples had higher glucose yields by 33% (*w*/*w*) and 35% (*w*/*w*), respectively, than the acid-pretreated samples. In this study, the alkali pretreatment method released more glucose than the acid method did for all Enset biomass samples; this was due to the compositional differences between each Enset biomass sample. Research has shown that the most effective pretreatment methods vary significantly depending on the type of biomass [66]. The alkali pretreatment method enhances cellulose digestibility, which makes it easier to remove lignin from the biomass than with the acid pretreatment method [68]; several lignocellulosic materials, such as corn stover, switchgrass, and Bermuda grass, have been successfully pretreated in this way [38]. However, the acid pretreatment method is primarily responsible for eliminating hemicellulosic materials from biomass and releasing sugars, such as xylose and arabinose, into the liquid stream [69]. In this study, we observed that between all of the samples, greater monomeric sugars were found in acid-pretreated than in alkali-pretreated liquids (Table S1 in Supplementary Materials), showing that the hemicellulose portion was more strongly solubilized than alkali-pretreated samples. Yet, following the washing process, the monomeric sugars were lost from the acid-pretreated samples. When comparing our findings with those for different biomasses from a previous study under similar alkaline conditions and enzymatic hydrolysis, 44.81 g/L glucose was found in switchgrass [38] and 48.68 g/L glucose in corn cobs [70] after 72 h, values that are comparable to the findings for most Enset biomass samples. This shows that Enset biomass could represent a potential raw material for biobutanol production. However, further investigations should be carried out to optimize the enzymatic hydrolysis process.

### 3.3. ABE Fermentation

**Figure 5:**
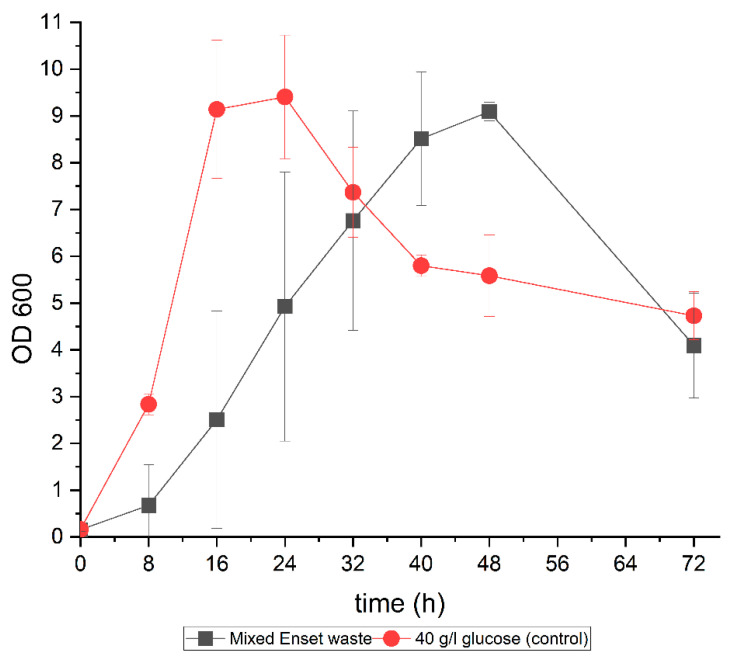
Growth profiles of *C. saccharoperbutylacetonicum DSM 14923* during fermentation of mixed Enset waste hydrolysate and pure glucose (40 g/L) as a control.

**Figure 6:**
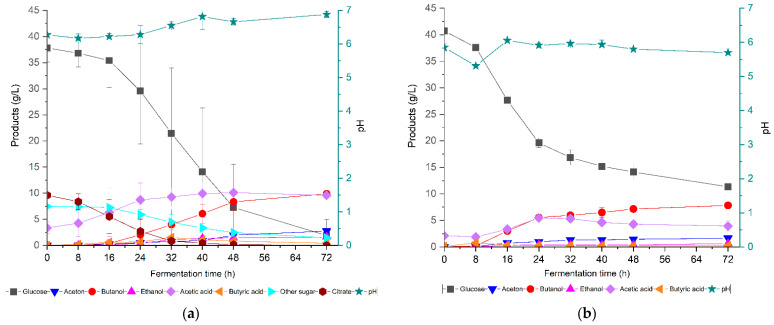
ABE fermentation using *C. saccharoperbutylacetonicum DSM 14923* from different carbon sources: (**a**) enzymatic hydrolysates of mixed Enset waste, pretreated with 2% (*w*/*w*) NaOH; (**b**) control medium with 40 g/L glucose.

ABE fermentation was carried out using the alkali-pretreated mixed Enset waste hydrolysate with *Clostridium saccharoperbutylacetonicum DSM 14923*. The fermentation results for the mixed Enset waste hydrolysate were compared to a control medium containing pure glucose (40 g/L) as the substrate. The growth profiles of the cells differed when the hydrolysate and pure glucose were used as different carbon sources, but they achieved the same maximum OD (Figure 5). Cell growth started 8 h faster in the control than in the hydrolysate medium, and the maximum OD_600_ of 9 was reached after 16 and 48 h, respectively. This could be due to the effect of the preculture medium since the preculture was grown on a glucose medium and it took a while for the cell to adapt to the mixed Enset waste hydrolysate medium. In addition, there might have been growth inhibition by citrate and other sugars in the mixed Enset waste hydrolysate medium (Figure 6a). However, after 32 h, the strain converted all of the citrate into acetic acid. Research has shown that during ABE fermentation, acetic acid helps increase the buffering capacity, prevent degeneration, and increase the CoA transferase activity [71].

Figure 6 shows the concentrations of ABE fermentation metabolites produced from alkali-pretreated mixed Enset waste hydrolysate and a control medium with 40 g/L glucose using *C. saccharoperbutylacetonicum DSM 14923*. The sugar consumption varied in fermentation depending on the initial sugar concentration; initially, the hydrolysate medium contained 37.8 g/L glucose, 7.5 g/L other sugars (xylose, mannose, and galactose), and 9.6 g/L citrate for the enzymatic hydrolysis process to maintain the pH. After 72 h of fermentation, only 1.9 g/L glucose and 1.5 g/L other sugar remained unused for the hydrolysate. Furthermore, it was observed in this study that all the citrate was consumed by the strain after 32 h. Similarly, after 72 h, 9.9 g/L butanol, 2.8 g/L acetone, and 1.6 g/L ethanol were obtained, achieving an ABE yield of 0.32 g/g and productivity of 0.2 g/(L h). After 16 h, 0.6 g/L butyric acid was produced, and this reached 1.5 g/L after 32 h. Overall, 3.4 g/L acetic acid was initially present in the hydrolysate medium and this gradually increased to 9.6 g/L after 32 h (Figure 6a). Acetic acid is produced in relatively high amounts, presumably due to the presence of citrate in the medium. It should be noted that after 32 h, the amount of acetic acid produced was quite small. Studies have shown that *clostridial* fermentation of citrate produces acetate and ethanol as the main products, along with negligible amounts of butanol and acetone [72].

During 72 h of fermentation on a glucose control medium, 28.7 g/L glucose was depleted by the culture and 7.8 g/L butanol, 1.6 g/L acetone, and 0.6 g/L ethanol were produced, resulting in an ABE yield of 0.25 g/g and a productivity of 0.14 g/(L h). In addition, during the first 8 h, 0.8 g/L butyric acid was detected, and a maximum of 5.5 g/L acetic acid was found at 24 h (Figure 6b). The ABE yield was calculated as the ratio between the total solvents produced and the sugar consumed, showing that the hydrolysate’s yield and thus its productivity were higher than those of the control fermentation, which could be due to the presence of sugars other than pure glucose. Yao et al. [44] reported that *C. saccharoperbutylacetonicum* is capable of utilizing glucose, cellobiose, xylose, arabinose, mannose, and galactose, but the rate depends on the type of sugar used. Additionally, in our research, once cell growth started, the rates of glucose consumption were similar in both cultures, but in the control medium, the cell growth and glucose consumption stopped at around 40 h due to the low pH. In the mixed Enset waste medium, meanwhile, the pH was higher due to the consumption of citrate during the first growth phase, which enabled the complete consumption of glucose. The ABE yield in this study supported the findings of previous studies carried out on the same strain with different biomasses, but the ABE productivity was lower than those found in previous studies [38,73]. Mixed Enset waste hydrolysate was utilized by *C. saccharoperbutylacetonicum* without extra detoxification or sugar supplementation. However, further research should be carried out to determine how we can maximize the yield and productivity.

## 4. Conclusions

This study found that Enset biomass parts contained high cellulose and low lignin, which contributed to producing a high level of glucose. In addition, low levels of inhibitory compounds were detected in all samples. The alkali-pretreatment method released more glucose from Enset biomass than the acid-pretreatment method. *C. saccharoperbutylacetonicum* utilized mixed Enset waste hydrolysate and produce 9.9 g/L butanol, 2.8 g/L acetone, and 1.6 g/L ethanol, achieving an ABE yield of 0.32 g/g and productivity of 0.2 g × L^−1^ × h^−1^). Enset biomass could represent an ideal candidate for biobutanol production. As part of our ongoing research, we are investigating the possibility of using biological pretreatment methods to reduce biomass loss during acid or alkaline pretreatments while protecting the environment at the same time.

## Figures and Tables

**Table 1:** Proximate and elemental analysis results for Enset biomass parts.

Analysis	LS1	LS2	LS3	LS4	UIS	LIS	UC	LC	LSP	EF	M	L
Proximate analysis (% dry weight, *w*/*w*)												
Ash	12.9	7.4	10.8	8.8	19.1	8.5	7.1	4.8	14.4	4.7	15.4	13.7
Moisture	7.4	9.7	9.4	10.3	7.8	9	10.2	10.6	7.7	7.3	6.5	5.7
Elemental analysis (% dry weight, *w*/*w*)												
C	37.8	38.5	37.9	38.2	37.3	39.1	38.4	38.4	37.9	41.2	37.1	42.8
H	5.5	6.1	6	6.2	5.2	5.9	6.2	6.2	5.5	6.1	5.2	5.7
N	0.5	0.5	0.8	0.6	2.5	1.2	0.6	0.6	0.7	0.3	1.2	3.3
O ^a^	43.3	47.5	44.5	46.2	35.9	45.3	47.7	50	41.5	47.7	41.1	34.5
Calorific value (CV) (MJ/kg)												
CV	14.4	15.02	14.7	14.8	14.7	15.3	14.9	14.98	14.62	16.14	14.28	17.4

Note: All experiments were done in triplicate, and the mean is reported here. ^a^ The percentage of O calculated from the difference between CHN and ash by assuming the sulfur content is small compared to oxygen [55].

**Table 2:** Lignocellulosic composition of Enset biomass parts.

Analysis (% Dry Matter)	LS1	LS2	LS3	LS4	UIS	LIS	UC	LC	LSP	EF	M	L
Cellulose	26.4	5.6	8	6.1	32	6.7	2.2	3.8 ^a^	34.1	67.1	40	20
Hemicellulose	18.6	10.2	9.5	6.6	20	20	6.2	11.4	15.7	15.6	19.7	27
Lignin	6.8	0.3	0.6	0.5	6.5	1.7	0.4	0.7	6.3	5.1	3.1	3.8

Note: All experiments were done in duplicate and the mean is reported here. ^a^ The sample was very difficult to grind, which increased the variance in the NDF measurements.

**Table 3:** Sugars and degradation products in liquid hydrolysate of Enset biomass parts after two-step acid hydrolysis. Values are given in % weight per weight dry Enset biomass part.

Compounds [% (*w*/*w*)]	Leaf Sheath Peel	Enset Fiber	Midrib	Mixed Enset Waste
Cellobiose	0.8 ± 0.3	2.5 ± 0.4	1.1 ± 0.2	1.3 ± 0.4
Glucose	56.4 ± 0.56	65.5 ± 4.73	39.1 ± 2.22	45.1 ± 0.21
Arabinose	2.4 ± 0.42	0.9 ± 0.16	3.1 ± 0.65	3.3 ± 0.04
Other sugar (xylose, mannose, and galactose)	11.4 ± 0.61	12.8 ± 0.68	10.5 ± 1.27	10.5 ± 0.84
Formic acid	2.0 ± 0.08	2.2 ± 0.23	2.1 ± 0.13	1.9 ± 0.12
Acetic acid	5.0 ± 0.46	6.7± 0.57	7.4 ± 0.55	9.0 ± 0.7
Furfural	0.8 ± 0.02	1.0 ± 0.04	0.4 ± 0.03	0.7 ± 0.00

Note: All experiments were done in triplicate, and the mean is reported here.

## Data Availability

Not applicable.

## Supplementary Materials

The following supporting information can be downloaded at https://www.mdpi.com/article/10.3390/bioengineering9040133/s1, Table S1: Monomeric sugars and degradation products in Enset biomass parts hydrolysate pretreated with different methods: (**a**) 2% (*w*/*v*) NaOH; (**b**) 2% (*v*/*v*) H_2_SO_4_.
