# Supplementary material for: Investigating the Processing Potential of Ethiopian Agricultural Residue Enset/Ensete ventricosum for Biobutanol Production
Source: Bioengineering (Basel). 2022 Mar 24;9(4):133. doi: 10.3390/bioengineering9040133 (PMC9025969; doi:10.3390/bioengineering9040133)
Supplement: Supplementary file 1 [file bioengineering-09-00133-s001.zip › bioengineering-1627960-supplementary.pdf]

bioengineering

MDPI

Supplementary Materials

Investigating the Processing Potential of Ethiopian Agricultural Residue Enset/*Ensete ventricosum* for Biobutanol Production

Nebyat Seid [1], Pia Griesheimer [2], and Anke Neumann [1,*]

1 Technical Biology, Institute of Process Engineering in Life Science 2, Karlsruhe Institute of Technology, 76131 Karlsruhe, Germany; nebyat.seid@kit.edu
2 Institute of Catalysis Research and Technology, Karlsruhe Institute of Technology, 76344 Eggenstein-Leopoldshafen, Germany; pia.griesheimer@kit.edu
* Correspondence: anke.neumann@kit.edu

Table S1. Monomeric sugars and degradation products in liquid hydrolysate Enset biomass parts after pretreated with different methods (**a**) alkali pretreatment (2% (*w*/*v*) NaOH); (**b**) acid pretreatment (2% (*v*/*v*) H_2SO_4).

**(a) alkali pretreatment (2% (*w*/*v*) NaOH)**				
**Analysis % (*w*/*w*)**	**Leaf sheath peel**	**Enset fiber**	**Midrib**	**Mixed Enset waste**
Cellobiose	0.04 ± 0.01	0	0.03 ± 0.00	0.07 ± 0.00
Glucose	0.83 ± 0.04	0.36 ± 0.03	1.44 ± 0.12	0.37 ± 0.01
Arabinose	0	0	0.05 ± 0.00	0
Other sugar (xylose, mannose, and galactose)	0.28 ± 0.02	0.17 ± 0.01	0.45 ± 0.06	0.28 ± 0.01
Formic acid	0.84 ± 0.16	0.59 ± 0.00	1.38 ± 0.04	1.03 ± 0.14
Acetic acid	5.41 ± 0.38	7.82 ± 0.01	11.01 ± 0.56	11.47 ± 0.50
**(b) acid pretreatment (2% (*v*/*v*) H_2SO_4)**				
**Analysis % (*w*/*w*)**	**Leaf sheath peel**	**Enset fiber**	**Midrib**	**Mixed Enset waste**
Cellobiose	0.88 ± 0.77	2.09 ± 0.02	1.28 ± 0.02	1.32 ± 0.03
Glucose	10.46 ± 1.27	2.33 ± 0.13	4.71 ± 0.12	7.82 ± 0.20
Arabinose	1.07 ± 0.20	0.38 ± 0.03	3.66 ± 0.09	2.58 ± 0.05
Other sugar (xylose, mannose, and galactose)	7.37 ± 0.39	9.38 ± 0.04	9.33 ± 0.17	7.15 ± 0.12
Formic acid	0	0	0.38 ± 0.05	0.27 ± 0.02
Acetic acid	4.46 ± 0.54	5.95 ± 0.09	7.72 ± 0.87	8.25 ± 0.27
